# Estrogen Attenuates the Growth of Human Papillomavirus-Positive Epithelial Cells

**DOI:** 10.1128/mSphere.00049-20

**Published:** 2020-03-18

**Authors:** Molly L. Bristol, Claire D. James, Xu Wang, Christian T. Fontan, Iain M. Morgan

**Affiliations:** a Virginia Commonwealth University, Philips Institute for Oral Health Research, School of Dentistry, Richmond, Virginia, USA; b VCU Massey Cancer Center, Virginia Commonwealth University, Richmond, Virginia, USA; Northwestern University

**Keywords:** HNSCC, HPV, cancer, estrogen, keratinocyte, transcription

## Abstract

Human papillomaviruses (HPVs) are small, double-stranded DNA viruses that are significant risk factors in the development of cancer, and HPV accounts for approximately 5% of all worldwide cancers. Recent studies using data from The Cancer Genome Atlas (TCGA) have demonstrated that elevated levels of estrogen receptor alpha (ERα) are associated with improved survival in oropharyngeal cancers, and these elevated receptor levels were linked with human papillomavirus-positive cancers (HPV+cancers). There has been a dramatic increase in HPV-related head and neck squamous cell carcinomas (HPV+HNSCCs) over the last 2 decades, and therapeutic options for this ongoing health crisis are a priority; currently, there are no antiviral therapeutics available for combatting HPV+cancers. During our TGCA studies on head and neck cancer, we had also discovered the overexpression of ERα in HPV+cancers. Here, we demonstrate that 17β-estradiol (estrogen) attenuates the growth/cell viability of HPV+cancers *in vitro*, but not HPV-negative cancer cells. In addition, N/Tert-1 cells (foreskin keratinocytes immortalized with human telomerase reverse transcriptase [hTERT]) containing human papillomavirus 16 (HPV16) have elevated levels of ERα and growth sensitivity after estrogen treatment compared with parental N/Tert-1 cells. Finally, we demonstrate that there are potentially two mechanisms contributing to the attenuation of HPV+ cell growth following estrogen treatment. First, estrogen represses the viral transcriptional long control region (LCR) downregulating early gene expression, including E6/E7. Second, expression of E6 and E7 by themselves sensitizes cells to estrogen. Overall, our results support the recent proposal that estrogen could be exploited therapeutically for the treatment of HPV-positive oral cancers.

**IMPORTANCE** Human papillomaviruses cause around 5% of all human cancers, yet there are no specific antiviral therapeutic approaches available for combatting these cancers. These cancers are currently treated with standard chemoradiation therapy (CRT). Specific antiviral reagents are desperately required, particularly for HPV+HNSCC whose incidence is increasing and for which there are no diagnostic tools available for combatting this disease. Using data from The Cancer Genome Atlas (TCGA), we and others determined that the estrogen receptor alpha (ERα) is overexpressed in HPV+HNSCC and that elevated levels are associated with an improved disease outcome. This has led to the proposal that estrogen treatment could be a novel therapeutic approach for combatting HPV+cancers. Here, we demonstrate that estrogen attenuates the growth of HPV+epithelial cells using multiple mechanisms, supporting the idea that estrogen has potential as a therapeutic agent for the treatment of HPV+HNSCC.

## Abstract (precis)

Human papillomaviruses cause around 5% of all human cancers, yet there are no specific antiviral therapeutic approaches available for combatting these cancers. These cancers are currently treated with standard chemoradiation therapy (CRT). Specific antiviral reagents are desperately required, particularly for HPV+HNSCC whose incidence is increasing and for which there are no diagnostic tools available for combatting this disease. Using data from The Cancer Genome Atlas (TCGA), we and others determined that the estrogen receptor alpha (ERα) is overexpressed in HPV+HNSCC and that elevated levels are associated with an improved disease outcome. This has led to the proposal that estrogen treatment could be a novel therapeutic approach for combatting HPV+cancers. Here, we demonstrate that estrogen attenuates the growth of HPV+epithelial cells using multiple mechanisms, supporting the idea that estrogen has potential as a therapeutic agent for the treatment of HPV+HNSCC.

## INTRODUCTION

Human papillomavirus (HPV) is the most common sexually transmitted infection in the United States, infecting nearly every sexually active person at some point in their lives (1–8). Of the high-risk HPVs known to cause cancers, human papillomavirus 16 (HPV16) is the most common genotype, accounting for 50% of cervical cancers and nearly 90% of HPV-related head and neck squamous cell carcinomas (HPV+HNSCCs) (4, 9, 10). The level of HPV-related HNSCCs has become an epidemic in the last decade, with more than half a million new cases per year worldwide (11). While prophylactic vaccines should be successful in preventing future HPV infections, there are currently no HPV-specific antiviral drugs to treat current HPV infections or HPV+HNSCCs.

A number of studies have implicated steroid hormones, including 17β-estradiol (estrogen), as cofactors in HPV carcinogenesis (12–17). For example, the estrogen receptor has been shown to play an important role in the development of cervical cancer in a K14-HPV16 E7 transgenic mouse model, where estrogen was determined to work as a cocarcinogen with E7 (14–16, 18, 19). However, the role of estrogen in the development of head and neck cancer in these transgenic mouse models has not been reported. In contrast to these results, studies demonstrate that high expression of estrogen receptor alpha (ERα) correlates with increased survival in HPV+HNSCC (20, 21). These reports suggest ERα as a diagnostic marker but also raise the possibility of using estrogen as a therapeutic for the treatment of HPV+HNSCC. In support of the potential therapeutic potential of estrogen for HPV-positive (HPV+) cancers, HeLa cells, an HPV18+ cervical cancer cell line, are extremely sensitive to estrogen treatment (22, 23). Given these recent reports, we investigated the ability of estrogen to regulate the growth of HPV+ cell lines.

Analysis of our The Cancer Genome Atlas (TCGA) data agreed with those of others; the ERα receptor was overexpressed in HPV+HNSCC compared with HPV-negative HNSCC (HPV−HNSCC), and higher expression predicted better overall survival (20, 21, 24). Here, we report that estrogen treatment results in growth attenuation of HPV16+HNSCC lines (SCC47 and UMSCC104) but does not significantly alter the growth of HPV-negative (HPV−) cancer cell lines. Previously we reported the transcriptional reprogramming of N/Tert-1 cells (foreskin cells immortalized by human telomerase reverse transcriptase [hTERT]) by HPV16 (N/Tert-1+HPV16) and demonstrate here that the growth of these cells is attenuated by estrogen while control parental N/Tert-1 cell growth was not affected by estrogen treatment. We also treated human tonsil keratinocytes that were immortalized by HPV16 (HTK+HPV16) and these were severely growth attenuated following estrogen treatment. In SCC47, UMSCC104, UMSCC152, N/Tert-1+HPV16 (clonal and pooled lines), and HTK+HPV16 cells treated with estrogen, a significant reduction of early gene RNA transcript levels, including E6 and E7, is observed. Using HPV16 LCR (the long control region that regulates transcription from the HPV16 genome) luciferase vectors, we demonstrate that estrogen can downregulate transcription from the HPV16 LCR. This downregulation has the potential to increase the p53 and pRb levels in the cells (the cellular targets for E6 and E7, respectively, that promote degradation of these tumor suppressors). However, while p53 levels were altered in SCC47 and UMSCC104 cells, it was not altered in other lines; similarly, pRb was significantly altered only in HeLa cells, indicating that the story may be more complex. While PARP1 cleavage was observed in SCC47, UMSCC152, and HeLa cells, it was not significantly altered in UMSCC104 cells, suggesting that growth attenuation is mediated by both apoptotic and nonapoptotic mechanisms, depending on the cell line. Finally, we treated N/Tert-1 cells expressing E6, E7, or E6+E7 (generated using retroviral transduction of the viral genes) with estrogen and demonstrate that expression of these viral oncoproteins by themselves results in growth attenuation of N/Tert-1 cells following estrogen treatment; however, this growth attenuation is delayed compared to N/Tert-1+HPV16 cells (25). Moreover, in these E6, E7, or E6+E7 cells, viral oncogene expression is not driven by the LCR, and the levels of the viral RNA transcript do not change following estrogen treatment. In conclusion, the results demonstrate that estrogen attenuates the growth of HPV16+ keratinocytes and HPV+ cancer cells and that there are potentially dual mechanisms for this attenuation: repression of viral transcription via targeting of the LCR and cellular reprograming of the host by E6/E7 that promotes the estrogen sensitivity. Our results support the idea that estrogen can be used as a potential therapeutic for the treatment of HPV+HNSCC. In further support of this idea, we demonstrate that estrogen plus radiation treatment of the HPV+HNSCC line SCC47 results in an additive attenuation of cell growth. No such effect was observed in the control HPV−HNSCC line, HN30.

## RESULTS

### Estrogen attenuates the growth of HPV16-positive head and neck cancer cell lines.

**FIG 1:**
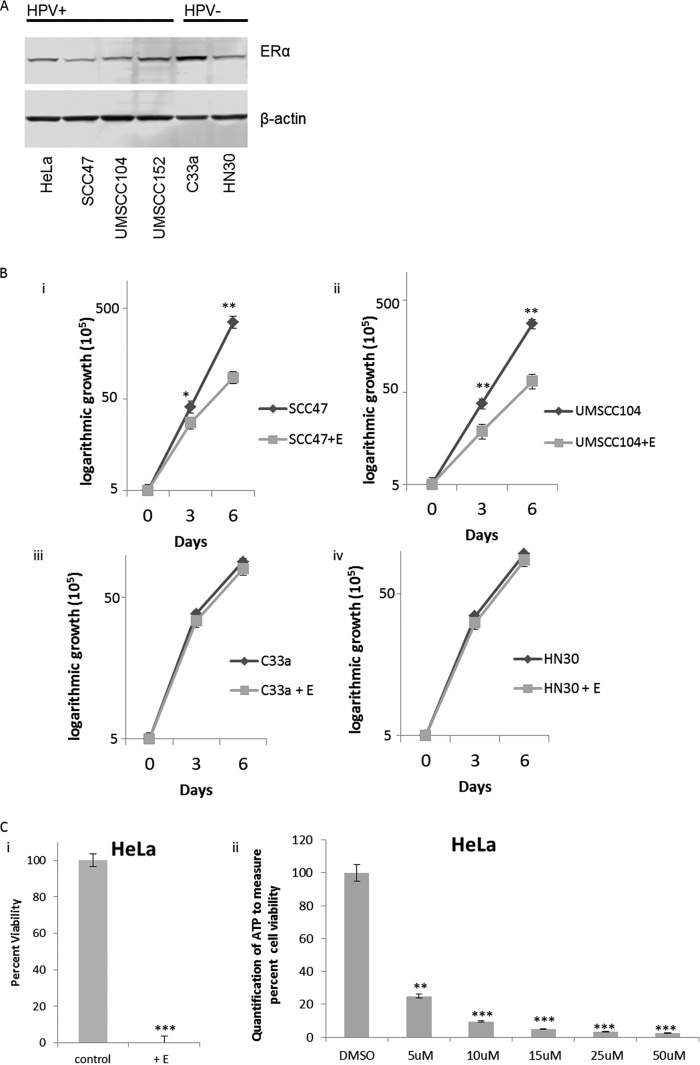
Estrogen attenuates the growth of HPV-positive (HPV+) or HPV-negative (HPV−) cancer cell lines. (A) Cervical cancer cell lines HeLa and C33a, as well as HNSCC cell lines SCC47, UMSCC104, UMSCC152, and HN30 were analyzed for their expression of the ERα and compared to the loading control β-actin. HPV status is indicated above the blots. Experiments were conducted in triplicate, and no significant correlation between HPV status and ERα expression was observed. (B) HPV+ SCC47 (i) and UMSCC104 (ii) cells and HPV− C33a (iii) and HN30 (iv) cells were seeded on day zero and grown in the presence of 15 μM estrogen (+E) or absence of estrogen. Cells were trypsinized and counted on days 3 and 6, and cell counts are presented on a logarithmic scale. Statistical differences in both SCC47 and UMSCC104 cells can be observed at both days 3 and 6. Values that are significantly different are indicated by asterisks as follows: *, *P* < 0.05; **, *P* < 0.001. No statistical difference is observed between treatments on day 3 or day 6 in C33a cells (iii) or HN30 cells (iv). Experiments were conducted in triplicate, and error bars are representative of the standard errors (SE). (C) (i) HeLa cells were grown in the presence or absence of 15 μM estrogen for 72 h, and then cells were counted for viability via trypan blue exclusion. (ii) Data are presented as percent viability at 48 h as measured by luciferase to monitor ATP via the Promega Cell Titer-Glo assay, over DMSO control. Experiments were conducted in triplicate, and error bars are representative of SE. **, *P* < 0.001; **, *P* < 0.001.

We have reported differential gene expression between HPV16+HNSCC and HPV−HNSCC using data from TCGA (24). We further analyzed this and observed that the ERα expression was increased in HPV16+HNSCC than in HPV−HNSCC; as we were doing these studies, two other reports were published demonstrating the increased expression of ERα in HPV+HNSCC (20, 21). Moreover, these studies demonstrated that increased levels of ERα predicted better survival, suggesting that this receptor may be of diagnostic significance and that estrogen could be a novel therapeutic for targeting HPV+HNSCC (20, 21). We investigated the protein expression levels of ERα in HPV-positive and -negative cancer cells (Fig. 1A). It is clear from this figure that any minor differences in protein expression of the ERα do not appear to be solely dependent on the HPV status of the cell line. Nevertheless, we proceeded to treat SCC47 and UMSCC104 (HPV16+HNSCC integrated and episomal, respectively), C33a (HPV-negative cervical cancer cell line), and HN30 (HPV−HNSCC) cells with estrogen and monitored cellular growth over a 6-day period (Fig. 1B). There was a significant attenuation of the growth with SCC47 (Fig. 1Bi) and UMSCC104 (Fig. 1Bii) following treatment with estrogen, but not with C33a (Fig. 1Biii) or HN30 (Fig. 1Biv). Likewise, the HPV18+ HeLa cervical cancer cells were also grown in the presence or absence of estrogen. Strikingly, all the HeLa cells appeared to be dead after 72 h of estrogen treatment (Fig. 1Ci) when we tried to observe HeLa cell growth in the presence or absence of estrogen, rendering cell growth observation impossible. To further analyze estrogen treatment in HeLa cells, the cells were treated with various doses of estrogen for 48 h, and subjected to a cell viability assay by monitoring ATP release via Cell Titer-Glo; as observed in Fig. 1Cii, estrogen significantly reduced HeLa cell viability at all doses tested. Recently, Li et al. also observed this phenomenon (23), which indicates that estrogen may provide a unique approach to attenuate the growth or to kill HPV+ cells.

**FIG 2:**
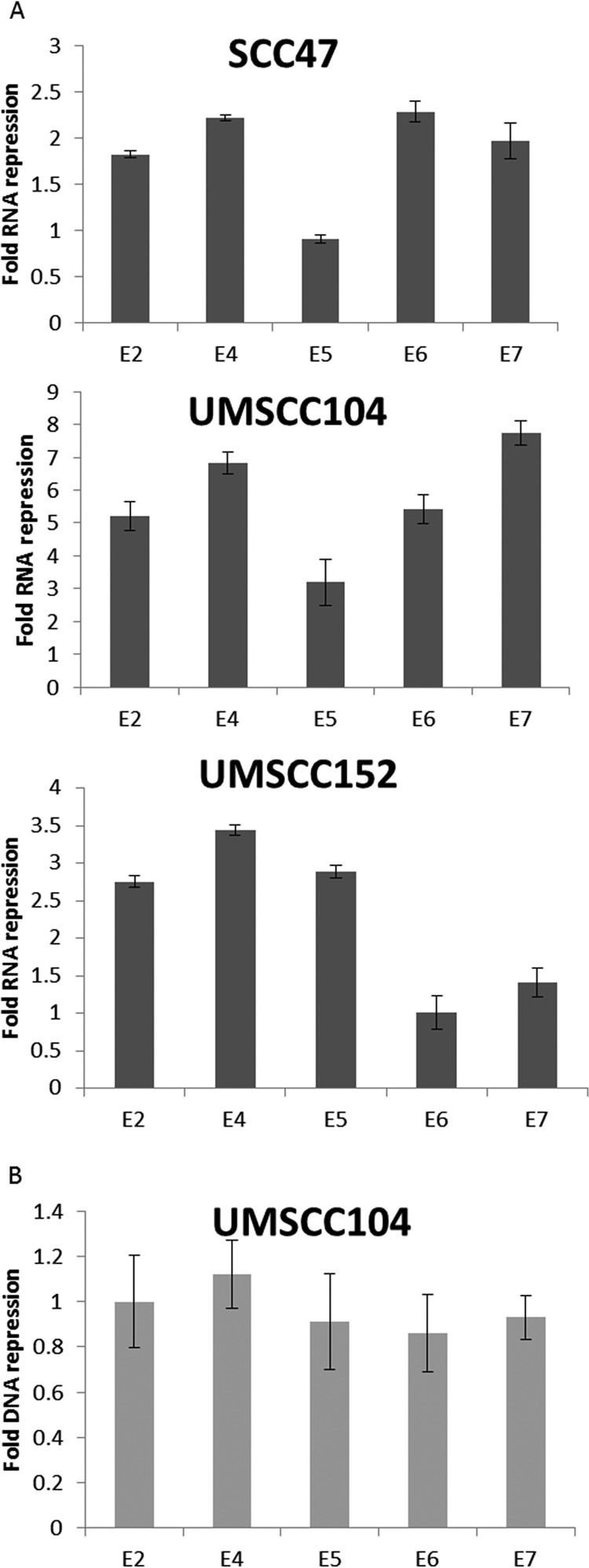
Estrogen significantly represses RNA expression of HPV16 early genes. (A) SCC47, UMSCC104, and UMSCC152 cells were grown in the presence or absence of 15 μM estrogen for 7 days. The cells were then harvested, and RNA expression levels were monitored via qPCR for E2, E4, E5, E6, and E7 and compared to the loading control GAPDH. Data are presented as fold repression calculated from ΔΔ*C_T_* calculated from the comparison of levels observed in control cells and further compared to GAPDH levels. (B) Cells were treated as described above for panel A, and DNA levels of E2, E4, E5, E6, and E7 were monitored via qPCR. Data are presented as fold repression calculated from ΔΔ*C_T_* calculated from the comparison of levels observed in control cells and further compared to GAPDH levels. No significant DNA changes were observed in any of the cell lines, and UMSCC104 data are presented as representative data. Experiments were conducted in triplicate, and error bars are representative of SE.

We further investigated whether estrogen treatment reduced the levels of HPV16 transcripts in these cells, as reduction of E6 and E7 levels has the potential to reactivate the p53 and pRb tumor suppressor pathways that would attenuate cellular growth. Figure 2A demonstrates that in SCC47, UMSCC104, and UMSCC152 (an HPV16+HNSCC line with a mixed population of integrated and episomal viral genomes), estrogen treatment for 7 days results in a significant reduction in viral RNA transcript levels. However, representative data from UMSCC104 cells show that there was no significant reduction of the viral DNA levels during this treatment (Fig. 2B). The results from Fig. 1 and 2 demonstrate that estrogen can selectively attenuate the growth of HPV16+HNSCC cell lines and reduce the viral transcript levels in these cells.

### An HPV16 isogenic model demonstrates that the presence of HPV16 imparts ERα upregulation and estrogen sensitivity.

**FIG 3:**
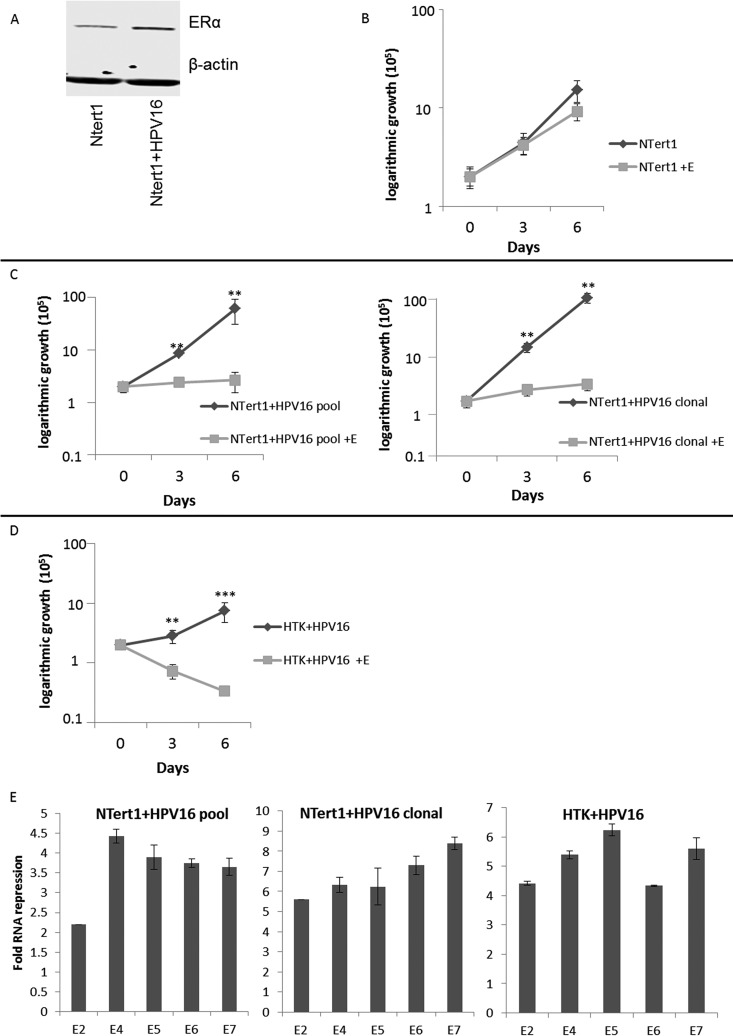
HPV16 confers estrogen sensitivity to N/Tert-1 cells. (A) Parental N/Tert-1 cell lines and our clonal N/Tert-1+HPV16 cell lines were analyzed for their overall ERα expression levels and compared to the loading control β-actin. (B to D) N/Tert-1 (B), N/Tert-1+HPV16 (pool and clonal) (C), and HTK+HPV16 (D) cells were seeded on day zero and grown in the presence or absence of 15 μM estrogen. Cells were trypsinized and counted on days 3 and 6, and cell counts are presented on a logarithmic scale. Statistical differences can be observed on both days 3 and 6 in all lines except the parental N/Tert-1 cells. **, *P* < 0.001; ***, *P* < 0.0001. Experiments were conducted in triplicate and error bars are representative of SE. (E) Pooled N/Tert-1+HPV16, clonal N/Tert-1+HPV16, and pooled HTK+HPV16 cells were grown in the presence or absence of 15 μM estrogen for 7 days. Cells were then harvested, and RNA expression levels were monitored via qPCR for E2, E4, E5, E6, and E7 and compared to the loading control GAPDH. Data are presented as fold repression calculated from ΔΔ*C_T_* calculated from the comparison of levels observed in control cells and further compared to GAPDH levels. Experiments were conducted in triplicate, and error bars are representative of SE.

Previously we reported on the development of an HPV16 life cycle model in N/Tert-1 cells (24, 25). In HPV16-infected N/Tert-1 (N/Tert-1+HPV16) cells, there is an increase in ERα expression over that in the parental N/Tert-1 cells (Fig. 3A). The comparison between N/Tert-1 parent cells and N/Tert-1+HPV16 cells allows an isogenic comparison of their response to external reagents. Figure 3B demonstrates that control N/Tert-1 cell growth was not significantly affected by estrogen treatment over a 6-day period; in comparison, both pooled and clonally generated N/Tert-1+HPV16 cells exhibited growth attenuation with estrogen treatment (Fig. 3C). We also investigated HPV16 host gene regulation in human tonsil keratinocytes immortalized by HPV16 (HTK+HPV16), and the growth of this cell line is severely attenuated by estrogen (Fig. 3D) (26). Expression of the viral RNAs were downregulated by estrogen treatment in both N/Tert-1+HPV16 and HTK+HPV16 cells (Fig. 3E). This is similar to the downregulation of viral RNA expression in the HPV16+HNSCC lines (Fig. 2A).

### Estrogen represses transcription from the HPV16 long control region.

**FIG 4:**
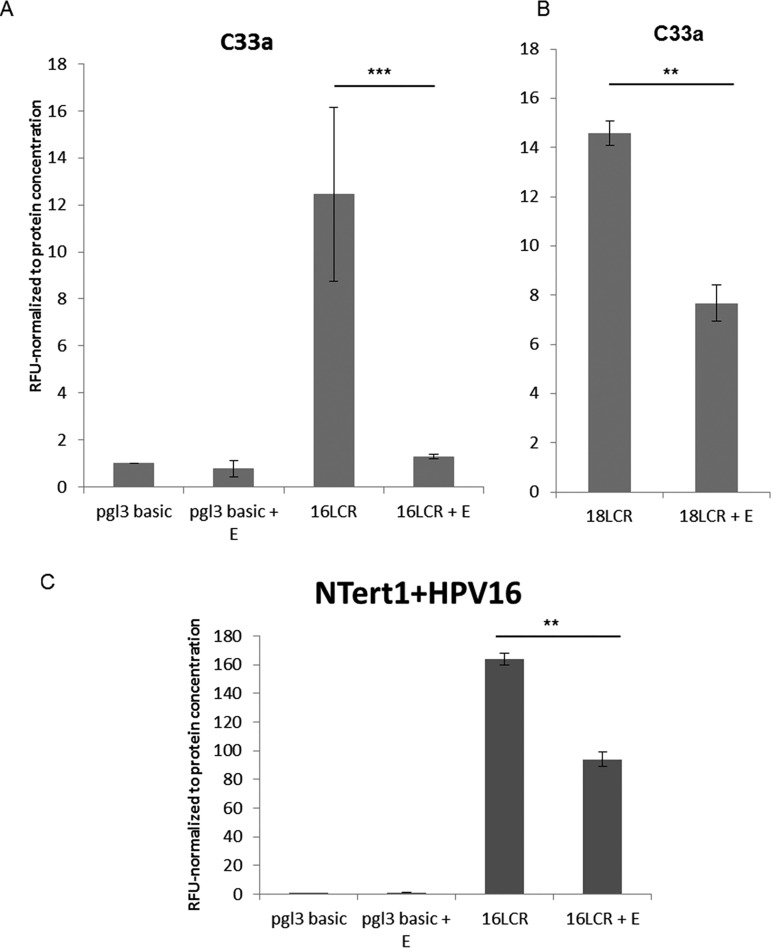
Estrogen significantly represses HPV16 and HPV18 LCR transcription. (A and B) C33a cells were transfected with 1 μg of pgl3 basic backbone (control), 1 μg 16LCR-pGL3 (A) or in 1 μg 18-LCR-pGL3 (B) and grown in the presence or absence of 15 μM estrogen. (C) N/Tert-1 cells were transfected with 1 μg of pgl3 basic backbone or 1 μg 16LCR-pGL3 and grown in the presence or absence of 15 μM estrogen. Forty-eight hours after transfection, a luciferase-based assay was utilized to monitor levels of LCR transcription. Data are presented as relative fluorescence units (RFU), normalized to total protein concentration as monitored by a standard bovine serum albumin (BSA) assay. **, *P* < 0.001; ***, *P* < 0.0001.

Figures 2 and 3 demonstrate that estrogen treatment of HPV16+ cells results in the repression of viral RNA expression. Transcription of HPV16 viral genes is regulated by the HPV16 long control region (HPV16 LCR), a region that is regulated by a number of host transcription factors. We constructed a reporter plasmid where luciferase gene expression is regulated by the HPV16-LCR (pHPV16-LCR-Luc), transfected this vector into C33a cells, and monitored transcription levels of the pHPV16-LCR-Luc via relative fluorescence units (RFU) in the presence or absence of estrogen. Estrogen treatment resulted in a significant reduction of luciferase expression (Fig. 4A), while expression from a control luciferase plasmid (pgl3 basic) was not affected by estrogen treatment. Because of the effects observed in HeLa cells (Fig. 1C), we sought to determine whether LCR repression was also observed in HPV18 in a previously described pHPV18-LCR-luc plasmid (27); similar significant repression of the HPV18 LCR was also observed (Fig. 4B). We conducted similar experiments in N/Tert-1 cells where estrogen treatment also significantly reduced luciferase activity in cells transfected with pHPV16-LCR-Luc (Fig. 4C) but did not reduce the control luciferase plasmid. The conclusion from Fig. 2 and 4 is that estrogen represses transcription from the HPV16 long control region to downregulate expression of early viral genes.

### Estrogen increases DNA damage and initiates apoptosis in some HPV+ cancer cells.

**FIG 5:**
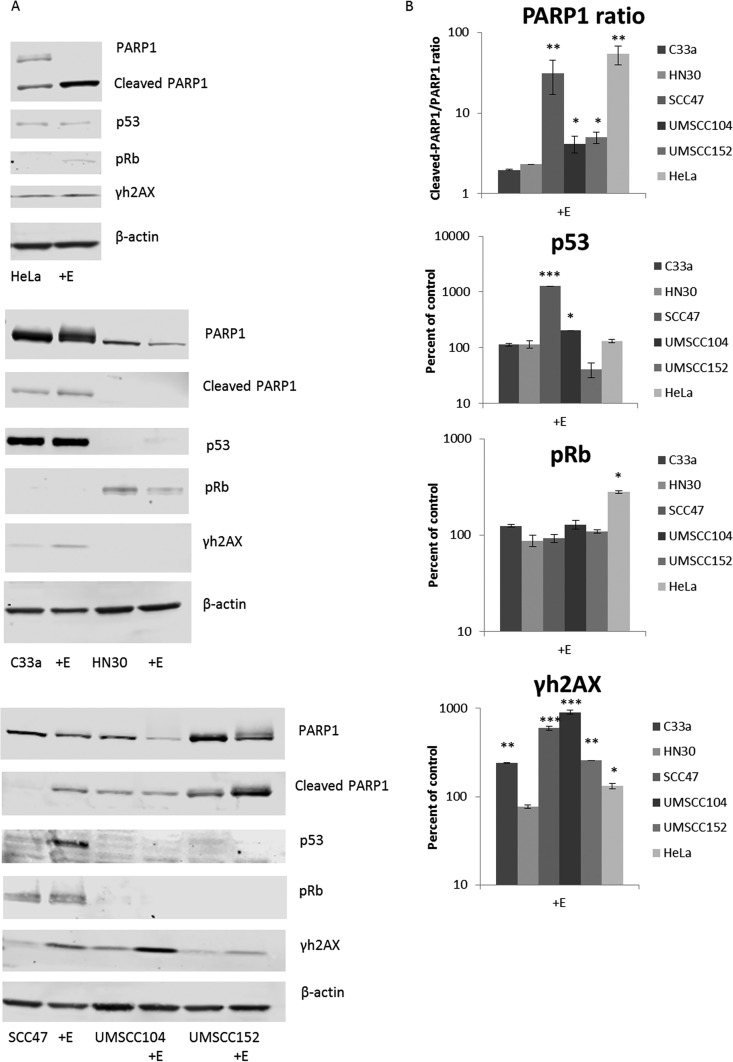
Estrogen alteration of protein expression in cancer cell lines. (A) HPV18+ HeLa cells (top panel), HPV− C33a and HN30 cells (middle panel), and HPV16+ SCC47, UMSCC104, and UMSCC152 cells (bottom panel) were grown in the presence or absence of 15 μM estrogen for 48 h. Cells were then lysed and analyzed via Western blotting for PARP1, cleaved PARP1, p53, pRb, and γh2AX. β-Actin was used as a loading control. (B) Densitometry analysis was compared from three independent experiments. For PARP1, the ratio of cleaved to noncleaved PARP1 is given, and the rest are presented in graphs as a percentage of the value for control cells. All values are normalized to the loading control and are in log scale.

Downregulation of E6 and E7 expression by estrogen could result in the elevation of p53 and pRb expression (their respective tumor suppressor targets) (28–41). Previously, studies have shown that when E2 is overexpressed in HPV-positive cervical cancer cells, it represses transcription from the viral LCR, and this repression reduces E6 and E7 levels and reactivates the p53 and pRb tumor suppressor proteins (31, 42–50). Moreover, E2 overexpression and loss of E6/E7 results in the elevation of p53 and pRb that allows for previously observed attenuation of growth in HeLa cells (23, 31, 45, 46, 48). Similarly, the results of our studies indicate that estrogen treatment represses transcription from the LCR to reduce expression of E6 and E7 levels. It has been well established that estrogen can induce DNA damage via the production of oxidative metabolites that cause DNA adducts or other oxidative damage, both *in vitro* and *in vivo* (51). We therefore analyzed the protein levels of p53 and pRb in our cancer cell lines in the presence or absence of estrogen, and we also monitored γh2AX as a marker for the initiation of the DNA damage response (52) and the ratio of cleaved PARP1/PARP1 as a marker for apoptosis. These Western blots are presented in Fig. 5A with accompanying densitometry analysis (Fig. 5B).

As expected, analysis of the response to estrogen in the sensitive HeLa cells revealed a significant increase in p53, pRb, γh2AX, and PARP1 cleavage (Fig. 5A, top panel), confirming the previously observed increase in apoptosis following estrogen in HeLa cells (23). Furthermore, analysis of the HPV− cancer cells reveals no dramatic alterations in p53, pRb, or PARP1 cleavage; however, there is a significant increase in γh2AX in C33a cells (Fig. 5A, middle panel). This increase in γh2AX reveals that estrogen is still initiating DNA damage; however, it appears that this damage alone is not sufficient to inhibit the growth of the C33a cells. Western blot analysis of our HPV+HNSCC lines reveals a less than clear-cut mechanism that allows for the reduction in cell growth observed (Fig. 5A, bottom panel). While all cells exhibited an increase in γh2AX and PARP1 cleavage, indicating that estrogen induces DNA damage that results in an increase of apoptosis, no significant alterations in pRb were observed in any of our HPV+HNSCC lines, and p53 was significantly increased only in SCC47 and UMSCC104 cells. It is possible that the low levels of E6 and E7 still present do not fully allow for alterations in p53 and pRb or that there is an increase in these tumor suppressors that cannot be detected by Western blotting. Therefore, the reactivation of these tumor suppressors following estrogen treatment does not fully explain the attenuation of cell growth in the HPV16+ cells.

### Expression of the viral oncogenes promotes delayed cell growth attenuation following estrogen treatment.

**FIG 6:**
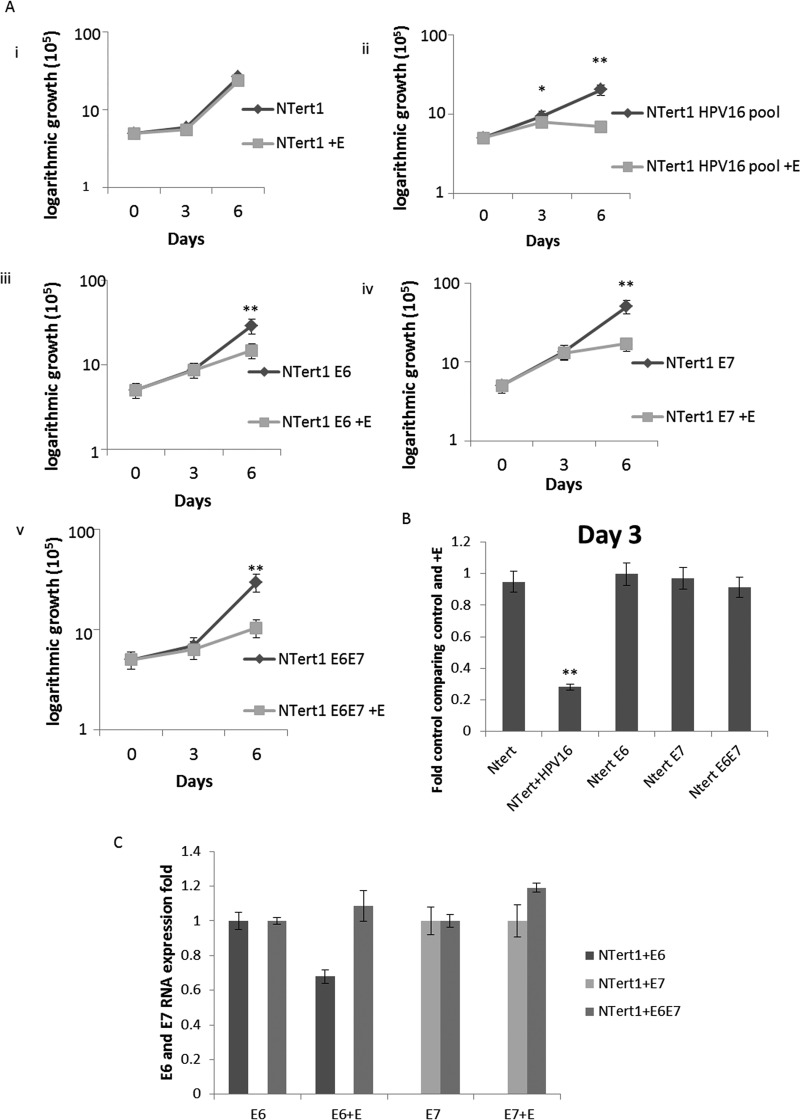
E6 and E7 expression by themselves sensitizes N/Tert-1 cells to estrogen. (A) N/Tert-1 (i), N/Tert-1+HPV16 (ii), N/Tert-1+E6 (iii), N/Tert-1+E7 (iv), and N/Tert-1+E6E7 (v) cells were seeded on day zero and grown in the presence or absence of estrogen. Cells were trypsinized and counted on daya 3 and 6, and cell counts are presented on a logarithmic scale. Statistical differences can be observed on both days 3 and day 6 in panel ii, but only on day 6 in panels iii to v. *, *P* < 0.05; **, *P* < 0.001. (B) Day 3 cell counts are compared as a percentage of control and normalized. Only N/Tert-1+HPV16 cells present a statistical difference at this time point. **, *P* < 0.001. (C) N/Tert-1+E6, N/Tert-1+E7, and N/Tert-1+E6E7 cells were analyzed for their RNA expression levels of E6 and E7 and compared to the loading control GAPDH. Data are presented as fold expression as calculated from ΔΔ*C_T_* calculated from the comparison of levels observed in control cells and further compared to GAPDH levels. No statistical differences were found.

We next investigated whether the transcriptional reprogramming of N/Tert-1 cells carried out by HPV16 oncogenes alone could sensitize cells to estrogen and attenuate cellular growth. To do this, we expressed E6 or E7 or E6+E7 in N/Tert-1 cells and further compared these cells to cells expressing the full HPV16 genome (N/Tert-1+HPV16); these E6, E7, and E6+E7 cell lines were generated using retroviral delivery and have been described previously (26, 53). Figure 6A demonstrates again that in N/Tert-1 control cells, estrogen treatment does not attenuate cellular growth (Fig. 6Ai), but the presence of the entire HPV16 genome promotes such attenuation (Fig. 6Aii). The presence of E6, E7, or E6+E7 resulted in growth attenuation following estrogen treatment (Fig. 6Aiii to v), although it was not observed on day 3, instead delaying the attenuation of cell growth that was observed with the entire HPV16 genome (comparison of day 3 is normalized and presented in Fig. 6B). As the expression of the E6 and E7 in panels iii to v of Fig. 6A is not driven by the viral LCR, but rather by retroviral sequences, we anticipated that the RNA levels of the oncogenes would not be regulated by estrogen. This is indeed the case; estrogen treatment did not alter E6 or E7 levels in the cells transduced with the retroviral vectors (Fig. 6C). Therefore, the growth attenuation of these cells following treatment with estrogen can be attributed to the expression of the viral oncoproteins, and likely due to the transcriptional reprogramming of these cells carried out by these proteins.

### Estrogen and radiation treatment of HPV-positive and -negative cancer cells.

**FIG 7:**
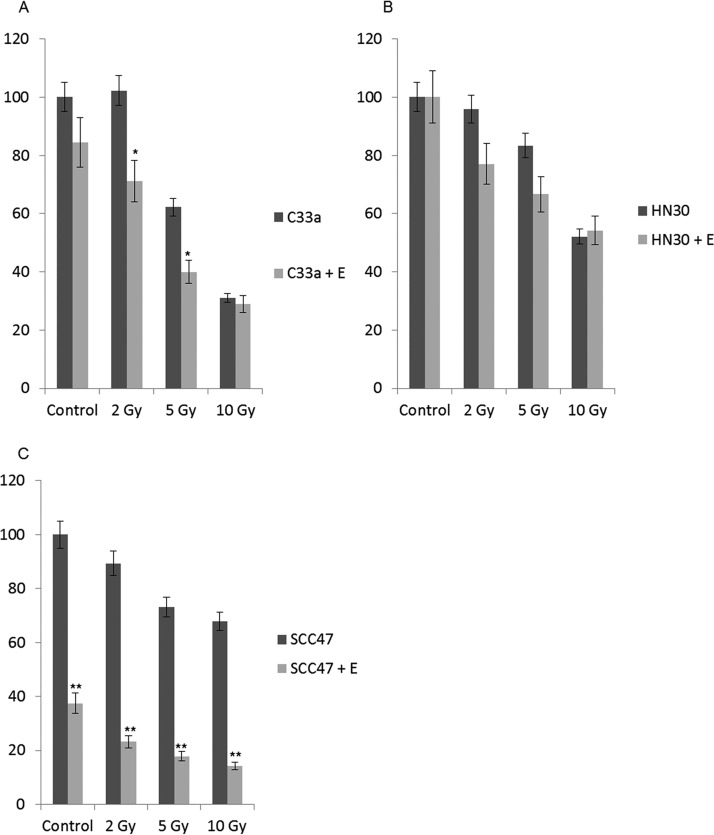
Estrogen enhances the response to radiation in SCC47 cells but not in C33a or HN30 cells. (A to C) C33a (A), HN30 (B), and SCC47 (C) cells were maintained in estrogen for 72 h. The indicated cells were then radiated with 2, 5, or 10 Gy radiation, and cells were trypsinized and counted by trypan blue exclusion for viability 72 h postirradiation. Data are presented as percent viability compared with untreated control cells. Experiments were conducted in triplicate, and error bars are representative of SE. *, *P* < 0.05; **, *P* < 0.001.

Radiation treatment is a standard of care therapy for HPV+HNSCCs. We treated C33a, HN30, and SCC47 cells with estrogen and then treated them with 2, 5, and 10 Gy of radiation to investigate whether estrogen can promote further response to this treatment. For C33a cells (Fig. 7A), estrogen did significantly attenuate cell growth at 2 Gy and 5 Gy, but not at 10 Gy. For HN30 cells (Fig. 7B), the presence of estrogen made no significant difference to the response to radiation treatment. For SCC47 cells, treatment with estrogen by itself attenuated cell growth, as shown in Fig. 1Bi. As observed in Fig. 7C, treatment with radiation did not have a dramatic effect on the growth of SCC47 cells. However, because SCC47 cells were highly sensitive to estrogen alone, the additive effect observed with estrogen and radiation led to ∼80% loss in cell viability even at 2 Gy radiation. Moreover, at 10 Gy, there was no significant attenuation of cell growth with the addition of estrogen in either C33a cells or HN30 cells, but a very significant repression was observed in SCC47 cells. This is promising and suggests that estrogen treatment may provide a unique opportunity to allow for increased responsiveness to radiation treatment in the clinic at reduced radiation doses for HPV+HNSCC.

## DISCUSSION

While the prophylactic vaccine should decrease the incidence of HPV in the coming decades, we currently lack antiviral treatments to target those already infected with the virus. Likewise, HPV-related HNSCCs are on the rise, and this oncogenic virus has bypassed tobacco as the main carcinogen in the oropharyngeal region (3, 11, 54). Despite HPV+HNSCCs and HPV−HNSCCs being very different both phenotypically and genotypically in terms of their pathological and molecular mechanisms of carcinogenesis and in their response to therapy, they are still treated the same in the clinic (55). It is therefore of particular interest to develop HPV-specific treatments for HPV+HNSCCs.

Analysis of TCGA data showed that the expression of the estrogen receptor alpha (ERα) was highly significantly upregulated in HPV16+HNSCCs versus HPV−HNSCCs (20, 21, 24). The ERα also decreased as stages advance, so we initially rationalized that estrogen may play a role in the early development of cancer. This differential expression of the ERα presented an opportunity to exploit a significant difference between HPV16+HNSCCs and HPV−HNSCCs and to possibly develop a specific targeted approach. Our initial hypothesis aligned with previous indications that estrogen and the ERα increase the risk of cervical cancer, and we further predicted that high doses of estrogen would initiate the DNA damage response (DDR) (14–16, 18, 19, 56–59).

On the basis of our previously published data, we further predicted that this increase in the DDR via estrogen would enhance HPV tumorigenicity and ultimately result in worse outcomes and disease progression (59). However, it soon became clear that our initial hypothesis was incorrect when HPV+ cells were specifically sensitized via estrogen treatment, while HPV− cells showed little to no response. We were also extremely surprised with the dramatic response to estrogen that we observed in HeLa cells, although recently published data confirm our observations (23). This recent study (23) utilizing HeLa cells as a model to analyze steroid signaling confirmed that these cells are particularly sensitive to estrogen. Li et al. showed that estrogen induced classical caspase-3-mediated apoptosis via a multistep molecular mechanism; however, this study did not take into account the HPV status of their cell model and may have missed an underlying viral mechanism by which estrogen was able to induce the cell death they observed (23). More specifically, HeLa cells are intrinsically dependent on the expression of E6 and E7 (60); if estrogen is able to reduce viral levels of these vital oncoproteins, this could contribute to the rapid progression to death observed for HeLa cells, although it is likely not the only mechanism.

While the expression of ERα was found to be upregulated in HPV+HNSCC, and via HPV expression in our N/Tert-1 model, we do not believe that the overall ERα expression level is the only reason that HPV+ cells are sensitive to estrogen. Among the cell lines we analyzed for estrogen sensitivity, the C33a cells had the highest protein level as observed by Western blotting (Fig. 1A), yet C33a cells showed little to no cell growth response to estrogen alone (Fig. 1Biii); however, estrogen did increase γh2AX, demonstrating that these cells are responsive to estrogen (Fig. 5A, middle panel), while only providing moderate sensitization to irradiation (Fig. 7A). It is likely that estrogen/HPV-specific interactions, both via the LCR and E6/E7, are responsible for the growth inhibition and cell death we observed in our HPV+ cell lines, not from DNA damage signaling alone. Nevertheless, the HPV upregulation of ERα likely ensures the ability of HPV-infected cells to respond to estrogen treatment. Further expanding this, high expression of the ERα alone, as observed in C33a cells, is not enough to confer estrogen sensitivity; HPV upregulation of the ERα in conjunction with HPV-specific estrogenic signaling initiates a complex signaling cascade to initiate estrogen sensitivity.

HPV+HNSCC is most commonly associated with males, found at a 4:1 higher ratio than observed in females (61). While estrogen is typically associated with females, men do in fact express appreciable levels of the estrogen receptors, and circulating estradiol levels in males are the same or higher than observed in postmenopausal women (62–65). With women having higher circulating estrogen levels for the majority of their life, this may suggest that estrogen is protective against HPV+HNSCC in women. Therefore, this could begin to explain some of the sex-related differences observed in the instances of HPV+HNSCC and presents an interesting observation for future studies.

It is not clear what control region in the HPV16 LCR is responsible for transcriptional repression following estrogen treatment. However, it has been shown that the ERα can interact with AP1 via c-Jun, and there are known AP1 binding sites in the HPV16 LCR that may mediate the response of this region to estrogen (66–72). This will be investigated in future studies.

Future studies determining the exact mechanism of the interaction between estrogen and HPV may provide additional opportunities to provide more specific targeted approaches to exploit this HPV-specific sensitization to estrogen for therapeutic gain in the treatment of HPV+cancers. Overall, our results indicate that estrogen may provide an approach that could be exploited therapeutically for the treatment of HPV+ epithelial cells.

## MATERIALS AND METHODS

### Cell culture.

C33a (ATCC), HN30 (generous gift from Hisashi Harada, VCU Philips Institute), SCC47 (Millipore), and HeLa (generous gift from Alison McBride, NIAID) cells were grown in Dulbecco’s modified Eagle’s medium (Invitrogen) and supplemented with 10% charcoal stripped fetal bovine serum (Gemini Bio-products). UMSCC104 (Millipore), and UMSCC152 (ATCC) cells were grown in Eagle’s minimum essential medium (EMEM) (Invitrogen) supplemented with nonessential amino acids (NEAA) (Gibco) and 10% charcoal stripped fetal bovine serum. N/Tert-1 cells and all derived cell lines, as well as HTK+HPV16 cells (a generous gift from Craig Meyers, Penn State University, Hershey) have been described previously (24, 25, 52, 59) and were maintained in keratinocyte-serum free medium (K-SFM; Invitrogen), supplemented with a 1% (vol/vol) penicillin-streptomycin mixture (ThermoFisher Scientific). All N/Tert-1 cells were also supplemented with 4 μg/ml hygromycin B (Millipore Sigma). For all cells not directly purchased from companies, the cell type was confirmed by Johns Hopkins or MD Anderson cell line authentication services, and the cells were maintained at 37°C in a 5% CO_2_–95% air atmosphere, routinely passaged every 3 or 4 days, and routinely monitored for mycoplasma.

### Trypan blue exclusion.

Cell supernatant was collected to allow for dead cell collection; attached cells were harvested by trypsinization and added to the cell supernatant. Total cells were stained with trypan blue, and viable cells were counted. The total number of cells was recorded, and the viable cell ratio was calculated.

### Plasmids.

pHPV16-LCR-Luc was generated by PCR amplification of the HPV16 LCR from W12 cells, introducing KpnI and BglIII restriction sites, and cloned into a pGL3 backbone (cloning primers listed below). The other plasmids utilized in these studies have been previously reported by others or used and described by this laboratory: pGL3 basic (73), pHPV18-LCR-Luc (27), HPV16 E6 (p6661 MSCV-IP N-HA only 16E6 [Addgene plasmid 42603; Peter Howley]), HPV16 E7 (p6640 MSCV-P C-FlagHA 16E7-Kozak [Addgene plasmid 35018; Peter Howley]), and HPV16 E6E7 (pLXSNE6E7 [Addgene plasmid 52394; Denise Galloway]).

The pHPV16-LCR-Luc cloning primers (Invitrogen) follow: for HPV16 LCR, forward 1 (position 7153), 5′-TCGAGGTACCGCTGTAAGTATTGTATGT-3′; forward 2 (position 7288), 5′-TCGAGGTACCATGCTTGTGTAACTATTG-3′; forward 3 (position 7423), 5′-TCGAGGTACCGTAGCGCCAGCGGCCATT-3′; forward 4 (position 7531), 5′-CGAGGTACCGTACGTTTCCTGCTTGCC-3′; forward 5 (position 7668), 5′-TCGAGGTACCCACTATGCGCCAACGCCT-3′; forward 6 (position 7737), 5′-CGAGGTACCGCATATTTGGCATAAGGT-3′; forward 7 (position 7873), 5′-CGAGGTACCCACATTTACAAGCAACTT-3′; and reverse (position 94), 5′-TCGAAGATCTGGGTCCTGAAACACTGCAGTTCTT-3′.

### Transfection assays and transcriptional activity.

The cells were plated at a density of 5 × 10^5^ in 100-mm dishes. The following day, plasmid DNA was transfected using the calcium phosphate method for C33a cells. N/Tert-1 cells were transfected utilizing Lipofectamine 2000 (according to the manufacturer’s instructions, ThermoFisher Scientific). Twenty-four hours after transfection, the cells were washed and supplemented with 15 μM 17β-estradiol. Forty-eight hours after transfection, cells were harvested utilizing Promega reporter lysis buffer and analyzed for luciferase using the Promega luciferase assay system. Concentrations were normalized to protein levels, as measured by the Bio-Rad protein assay dye, and relative fluorescence units were measured using the BioTek Synergy H1 hybrid reader. Experiments were performed in triplicate.

### Western blots.

Cells were trypsinized, washed twice with phosphate-buffered saline (PBS), pelleted, and resuspended in 200 μl of lysis buffer (0.5% Nonidet P-40, 50 mM Tris [pH 7.8], 150 mM NaCl) supplemented with a protease inhibitor mixture (Roche Molecular Biochemicals). The cell and lysis buffer mixture was incubated on ice for 30 min and centrifuged for 10 min at 18,000 × *g* at 4°C, and supernatant was collected. Protein levels were determined utilizing the Bio-Rad protein assay. Equal amounts of protein were boiled in 4× Laemmli sample buffer (Bio-Rad). Samples were then loaded onto a 4 to 12% gradient gel (Invitrogen), run at 120 V for ∼2 h, and transferred at 100 V for 1 h onto nitrocellulose membranes (Bio-Rad) using the wet blot method. The membrane was then blocked in Odyssey blocking buffer (diluted 1:1 with PBS), at room temperature for 1 h. After the membrane was blocked, it was probed with the following antibodies diluted in blocking buffer, and incubated overnight (O/N) at 4°C: phospho-histone H2A.X rabbit (catalog no. 9718S; Cell Signaling) diluted 1:1,000, β-actin mouse (sc-81178; Santa Cruz) diluted 1:2,000, ERα rabbit (ab32063; AbCam) diluted 1:1,000, p53 mouse (catalog no. 2524S; Cell Signaling) diluted 1:1,000, pRb mouse diluted 1:1,000 (catalog no. 9309S; Cell Signaling), PARP1 mouse (sc-8007; Santa Cruz) diluted 1:1,000, and cleaved-PARP1 rabbit (catalog no. 9541S; Cell Signaling) diluted 1:1,000. Following incubation with primary antibody, the membrane was washed with 0.01% PBS-Tween wash buffer before probing with Odyssey secondary antibody diluted 1:20,000, goat anti-mouse IRdye 800cw, goat anti-rabbit IRdye 680cw for 1 h at room temperature. The membrane was then washed in 0.01% PBS-Tween before infrared scanning using the Odyssey Li-Cor imaging system, which was also used to perform densitometry analysis. Experiments were performed in triplicate.

### SYBR green quantitative reverse transcription-PCR (qRT-PCR).

At the time of harvest, cells were washed twice with phosphate-buffered saline. RNA was immediately isolated using the SV total RNA isolation system (Promega) following the manufacturer’s instructions. Two micrograms of RNA was reverse transcribed into cDNA using the high-capacity reverse transcription kit (Applied Biosystems). cDNA and relevant primers were added to PowerUp SYBR green master mix (Applied Biosystems), and real-time PCR was performed using 7500 Fast real-time PCR system (Applied Biosystems). Results shown are the average values from three independent experiments with the relative quantity of genes determined by the ΔΔ*C_T_* method normalized to the endogenous control gene glyceraldehyde-3-phosphate dehydrogenase (GAPDH).

The primers (Invitrogen) used follow: for GAPDH, 5′-GGAGCGAGATCCCTCCAAAAT-3′ (forward) and 5′-GGCTGTTGTCATACTTCTCATGG-3′ (reverse); for E2, 5′- TGGAAGTGCAGTTTGATGGA-3′ (forward) and 5′-CCGCATGAACTTCCCATACT-3′ (reverse); for E4, 5′-GGCACCGAAGAAACACAGAC-3′ (forward) and 5′-AATCCGTCCTTTGTGTGAGC-3′ (reverse); for E5, 5′-CACAACATTACTGGCGTGCT-3′ (forward) and 5′-ACCTAAACGCAGAGGCTGCT-3′ (reverse); for E6, 5′-AATGTTTCAGGACCCACAGG-3′ (forward) and 5′-GCATAAATCCCGAAAAGCAA-3′ (reverse); for E7, 5′-CCGGACAGAGCCCATTACAAT-3′ (forward) and 5′-ACGTGTGTGCTTTGTACGCAC-3′ (reverse).

### CellTiter-Glo protocol for measuring cellular ATP.

A total of 2,000 cells were plated in 200 μl medium in clear-bottom black 96-well plates (catalog no. 655090; Greiner Bio One). The following day, the medium was removed from cells and replaced with 200 μl medium containing different concentrations of 17β-estradiol. Cells were then incubated for 48 h. Afterwards, 25 μl of reconstituted CellTiter-Glo luminescent cell viability reagent (catalog no. G7571; Promega) was added to each well and incubated for 5 min. Luminescence readings were taken using the BioTek Synergy H1 hybrid reader. Viability percentages were calculated by normalizing to the readings for dimethyl sulfoxide (DMSO)-treated cells, utilizing wells containing only medium as blanks for a control. DMSO wells were normalized to 100%.

### Radiation.

Cells were exposed to gamma irradiation (γ-IR) using a ^137^Cs irradiator. Radiation treatment consisted of a single dose of irradiation at 2, 5, or 10 Gy. In our studies, cells were exposed to estrogen for 72 h before irradiation. After irradiation, cells were washed once with PBS, and medium was replaced. Estrogen was then maintained on the indicated cells for an additional 72 h before cells were trypsinized and counted to determine cell viability.
